# A Randomized Trial of an Early Measles Vaccine at 4½ Months of Age in Guinea-Bissau: Sex-Differential Immunological Effects

**DOI:** 10.1371/journal.pone.0097536

**Published:** 2014-05-16

**Authors:** Kristoffer Jarlov Jensen, Mia Søndergaard, Andreas Andersen, Erliyani Sartono, Cesario Martins, May-Lill Garly, Jesper Eugen-Olsen, Henrik Ullum, Maria Yazdanbakhsh, Peter Aaby, Christine Stabell Benn, Christian Erikstrup

**Affiliations:** 1 Research Center for Vitamins and Vaccines (CVIVA), Bandim Health Project, Statens Serum Institut, Copenhagen, Denmark; 2 Department of Cardiovascular and Renal Research, Faculty of Health Sciences, University of Southern Denmark, Odense, Denmark; 3 Bandim Health Project, INDEPTH Network, Bissau, Guinea-Bissau; 4 Leiden University Medical Center, Leiden, The Netherlands; 5 Clinical Research Centre, Copenhagen University Hospital, Hvidovre, Denmark; 6 Department of Clinical Immunology, Rigshospitalet, Copenhagen, Denmark; 7 OPEN, Institute of Clinical Research, University of Southern Denmark/Odense University Hospital, Odense, Denmark; 8 Department of Clinical Immunology, Aarhus University Hospital, Aarhus, Denmark; Aeras, United States of America

## Abstract

**Background:**

After measles vaccine (MV), all-cause mortality is reduced more than can be explained by the prevention of measles, especially in females.

**Objective:**

We aimed to study the biological mechanisms underlying the observed non-specific and sex-differential effects of MV on mortality.

**Methods:**

Within a large randomised trial of MV at 4.5 months of age blood samples were obtained before and six weeks after randomisation to early MV or no early MV. We measured concentrations of cytokines and soluble receptors from plasma (interleukin-1 receptor agonist (IL-1Ra), IL-6, IL-8, IL-10, tumor necrosis factor (TNF)-α, monocyte chemoattractant protein (MCP)-1, soluble urokinase-type plasminogen activator receptor), and secreted cytokines (interferon-γ, TNF-α, IL-5, IL-10, IL-13, IL-17) after *in vitro* challenge with innate agonists and recall antigens. We analysed the effect of MV in multiple imputation regression, overall and stratified by sex. The majority of the infants had previously been enrolled in a randomised trial of neonatal vitamin A. *Post hoc* we explored the potential effect modification by neonatal vitamin A.

**Results:**

Overall, MV versus no MV was associated with higher plasma MCP-1 levels, but the effect was only significant among females. Additionally, MV was associated with increased plasma IL-1Ra. MV had significantly positive effects on plasma IL-1Ra and IL-8 levels in females, but not in males. These effects were strongest in vitamin A supplemented infants. Vitamin A shifted the effect of MV in a pro-inflammatory direction.

**Conclusions:**

In this explorative study we found indications of sex-differential effects of MV on several of the plasma biomarkers investigated; in particular MV increased levels in females, most strongly in vitamin A recipients. The findings support that sex and micronutrient supplementation should be taken into account when analysing vaccine effects.

**Trial Registration:**

clinicaltrials.gov number NCT 00168545

## Introduction

Routine childhood vaccines may have non-specific effects on mortality, i.e. effects beyond protection against the targeted disease [1], [2]. The standard titre measles vaccine (MV) seems to reduce all-cause child mortality considerably more than can be explained by prevention of measles infection. This non-specific beneficial effect of MV has been most pronounced in females [2]–[4]. Since the high child mortality in low-income countries is mainly caused by infectious diseases [5], it is plausible that MV modulates the child’s immune system to reduce susceptibility to unrelated infections.

Only very few studies have addressed the immunological background for the non-specific effects of MV. One study indicated that the non-measles specific cellular responses may transiently decrease within the first few weeks after MV [6], whereas another study reported higher Th1 cytokine responses and differentially expressed cellular activation markers 6 weeks after MV [7]. However, the possible immunological background for the sex-differential non-specific effects of MV still remains to be studied.

We conducted a randomised trial of early MV at 4.5 months of age at the Bandim Health Project (BHP) in Guinea-Bissau. The primary outcome of the main trial was mortality [2]. Overall, in the per-protocol analysis early MV was associated with an all-cause mortality rate ratio (MRR (95% confidence interval)) of 0.71 (0.50–1.00), strongest in females. Unexpectedly, the main trial found a significant interaction between early MV and neonatal vitamin A supplementation. Between 4.5 and 9 months of age where the trial compared measles vaccinated versus controls who had all three diphtheria-tetanus-pertussis vaccinations (DTP3) as their most recent vaccination, the overall MRR between 4.5 and 9 months of age was 0.33 (0.13–0.86) for children who had not received vitamin A, whereas there was no effect of MV for children who had received vitamin A [2].

Within the randomised trial we conducted a nested immunological subgroup study to investigate the overall and sex-specific effects of early MV on the responses of interferon (IFN)-γ, tumor necrosis factor (TNF)-α, interleukin (IL)-5, IL-10, IL-13, IL-17 to *in vitro* stimulation with innate agonists and recall antigens, and the plasma levels of IL-8, IL-1 Receptor antagonist (Ra), monocyte chemoattractant protein (MCP)-1, TNF-α, IL-10, IL-6 and soluble urokinase-type plasminogen activator receptor (suPAR) levels. We also investigated the potential modifying effect of vitamin A on the response to MV.

## Materials and Methods

### Study Design

The protocol for this trial and supporting CONSORT checklist are available as supporting information; see Checklist S1 and Protocol S1. The main trial has been described in detail elsewhere [2]. Briefly, 6,648 children who had received all three scheduled doses of diphtheria-tetanus-pertussis (DTP) vaccines were randomised at 4.5 months of age to receive an early MV (arm 1, one third of the children) or to follow the recommended vaccination schedule with MV at 9 months of age (arm 2+3, two thirds of the children). Children vaccinated at 4.5 months were re-vaccinated at 9 months of age. The vaccine used was a standard titre Edmonston-Zagreb measles vaccine (Serum Institute of India, Pune, India, batch number 2360).

According to the national childhood vaccination program, normal healthy infants received oral polio vaccine at birth, 6, 10, and 14 weeks of age; BCG at birth; DTP at 6, 10, and 14 weeks of age. Information about vaccines was recorded on a personal health card that was seen at regular visits by our field team.

At the time of the MV trial a vitamin-A-at-birth trial was on-going, where children were randomised to receive 25,000 IU vitamin A, 50,000 IU vitamin A, or placebo at birth.

The immunological subgroup study aimed to include 400 children between January and September 2006. During this period all children randomised to receive the early MV at 4.5 months of age (arm 1) and half of the children who did not receive early measles vaccine before 9 months (arm 2+3, randomly selected by block randomisation with 24 envelopes per bag) were invited to participate in the present immunological sub-study.

We had some minor deviations including 1) during the first 2 weeks of the study all children from arm 2+3 were enrolled; 2) a few children from arm 2+3 were erroneously bled in spite of randomisation to no blood sampling; 3) during short intermittent periods we did not invite all children due to limited capacity.

Children were eligible for inclusion into the immunological study until 6 months of age. Informed consent was obtained from the mother of the child by signature or fingerprint if the mother was illiterate. If the mother was not alive, the informed consent was obtained from the guardian of the child, which by tradition in Bissau usually is the grandmother. Provided written informed consent the children were assessed for enrolment. The weight, length and mid-upper-arm-circumference were measured. An axillary temperature was obtained and the mother was interviewed regarding background factors and current symptoms of her child by a field assistant. A physician examined the children. To avoid that acute illness influenced plasma biomarkers and stimulated cytokine production, children with the following characteristics were excluded from the immunological study even if they were included in the main trial: current fever or diarrhoea reported by the mother; an axillary temperature above 37.5°C; a respiratory rate at 60/minute or above; or current infection diagnosed by the physician.

A few children in each group were erroneously bled even though they fulfilled the exclusion criteria, because the lab technician overlooked that the symptom had been noted. We analysed all obtained blood samples for plasma biomarkers, irrespective of the child presenting with symptoms at baseline. For the analysis of the *in vitro* cytokine production, however, we adhered to the exclusion criteria.

All children were invited back for a post-vaccination blood sample 6 weeks later. A six-week time-span between vaccination and follow-up was chosen to minimize the impact of acute direct effects of the vaccine since we attempted to study the non-specific effects of the vaccine. If not presenting at the follow-up, children were called once a week until 10 weeks post-inclusion. At follow-up a similar examination was performed, this time not excluding anyone.

### Blood Sampling

Blood samples were obtained at baseline and at follow-up 6 weeks later from January 31 through October 31, 2006. Due to problems with obtaining venous blood samples and observed differences in cytokine levels between venous and capillary blood samples [8], the present study only included children from whom we had a capillary sample. Prior to bleeding, the skin was wiped with an antiseptic swab. Capillary blood was obtained by finger-prick into an EDTA-coated tube and a heparinised tube, respectively. The EDTA-coated tube was kept cold until plasma separation, whereas the heparinised tube was kept at ambient temperature until stimulation. Maximum time span from bleeding to blood processing in the laboratory was 3 hours. The EDTA blood was centrifuged at 3,500 rpm for 10 minutes, and the retrieved plasma was stored at –80°C until analysis. For all children a blood film was microscopically inspected for malaria parasitemia; no child had parasitemia.

### Full Blood Cultures

The *in vitro* stimulation assay was performed as previously described [9] with minor modifications in respect to the stimulation panel. Briefly, heparinised blood was diluted 1∶10 with supplemented RPMI-1640 medium. Stimulations were LPS (1 ng/ml) [a Toll-like receptor (TLR) 4 agonist], (S)-(2, 3-bis (palmitoyloxy)-(2-RS)-propyl)-N-palmitoyl-(R)-Cys-(S)-Ser-(S)-Lys4-OH, trihydrochloride (PAM3Cys) (100 ng/ml, Cayla-InvivoGen Europe, Toulouse, France) [a TLR1-2 agonist], phytohaemagglutinin (PHA) (2 µg/ml) [a T cell mitogen], purified protein derivative (PPD) of *Mycobacterium tuberculosis* (10 µg/ml) and tetanus toxoid (TT) (1.5 Lf/ml). Since the children were BCG and DTP vaccinated prior to enrolment, stimulation with PPD and TT were expected to trigger a recall response, PHA non-specifically elicits T cell proliferative responses, while stimulations with LPS and PAM trigger the innate response via TLR-4 and TLR-1/2, respectively. Supernatants were harvested after 24 hours for LPS and after 72 hours for PHA, PPD, TT and PAM. The supernatants were stored at minus 80°C until analysis.

### Measurement of Biomarkers

*In vitro* samples and plasma samples were analysed in Leiden, The Netherlands and Copenhagen, Denmark, respectively, both on the Luminex platform (Luminex 100, Luminex Corp., Austin, TX, USA). The *in vitro* stimulation assay was slightly modified from a previous study [9], with the following analytes (lower limit of detection (LLD) given in parenthesis): TNF-α (10 pg/ml), IFN-γ (5 pg/ml), IL-5 (3 pg/ml), IL-10, IL-13 (10 pg/ml) and IL-17 (10 pg/ml) (BioSource, Camarillo, CA, USA). In plasma, TNF-α (5 pg/ml), IL-10 (5 pg/ml), IL-6 (7 pg/ml), MCP-1 (10 pg/ml), IL-8 (2.8 pg/ml), IL-1Ra (30 pg/ml) were measured (Fluorokine MAP Multiplex Human Cytokine Panel A, R&D Systems, Minneapolis, MN, USA). Concentrations of suPAR (0.1 ng/ml), a marker of inflammation [10], were measured from plasma by enzyme-linked immunosorbent assay (ELISA) (suPARnostic, ViroGates, Birkerød, Denmark). Measurements below the LLD were referred to as non-detectable (ND). Baseline and follow-up samples from each child were measured on the same assay plate.

### Statistical Methods

The study was the first of its kind, making power calculations difficult. Our study was explorative and was performed to generate hypotheses rather than to test a specific hypotheses. Biomolecule distributions were summarized by geometric means (GM) and compared between the two randomization groups by geometric mean ratios (GMR). GMRs were obtained as anti-logged coefficients from linear regression on the log-concentrations. For several of the biomarkers a proportion of measurements were below the LLD of the assay. Censoring from these non-detectable (ND) measurements was handled by multiple imputations (MI) [11], [12] using *mi impute chained* combined with *intreg*. A burn-in of 5 iterations was used before 20 imputations were drawn. The final estimates were obtained from the imputed datasets by *mi estimate*. For distributions with >50% ND measurements, the proportion of detectable samples (>LDD) were analysed by Poisson regression resulting in proportion ratios (PR). GMRs and PRs were reported with 95% confidence intervals (CIs). A GMR or PR >1 may be interpreted as a positive MV effect while a GMR or PR <1 represents a negative MV effect.

Measurements of IL-13, IL-17 and IL-5 after *in vitro* stimulation were non-normally distributed with left-skewed tails. To analyse these outcome with an alternative model to MI and Poisson regression, a non-parametric median regression analysis was performed on the follow-up sample for the effect of MV.

For all outcomes the presence of interactions between vaccination and sex was tested. In the light of the mortality findings [2] we performed a *post hoc* analysis, examining in each sex the effects of MV depending on whether neonatal vitamin A had been given or not.

To address the potential caveat imposed by multiple comparisons, the p-values for the estimates of the MV effect on the plasma biomarker outcomes were adjusted for multiple test-procedures using the method by Holland [13].

All estimates were adjusted for baseline values and series of Luminex measurements. Analyses were performed with STATA 12 (Statacorp LP, College Station, TX).

### Ethics Statement

The MV trial including the immunological study and the consent procedure was approved by the National Committee on Health Ethics of The Ministry of Health in Guinea-Bissau, and the Danish Central Research Ethical Committee gave its consultative approval. The present subgroup study was registered with clinicaltrials.gov, number NCT 00168545.

## Results

**Figure 1:**
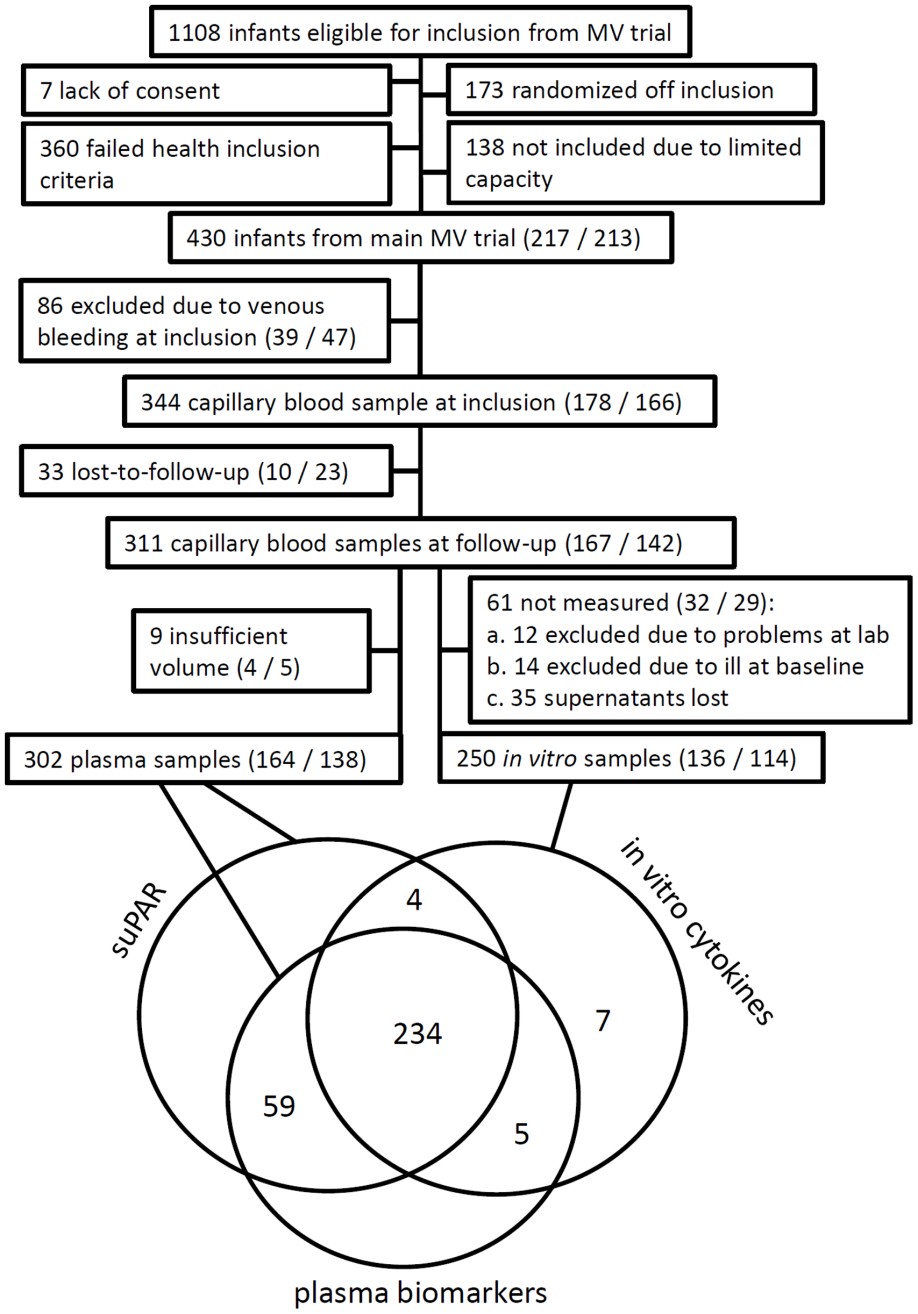
Flow chart of the immunological study. Numbers in parentheses designates group assignment, early MV or control group, respectively.

In total, information of one or more biological parameters in a paired baseline and follow-up sample was available for 309 infants, of which 167 (54%) were allocated to early MV (Figure 1); *in vitro* cytokine production after whole-blood stimulations was available from 250 infants, and plasma from 302 infants. There were no statistically significant differences between the intervention and control groups with regard to background characteristics (Table 1). Of the infants from the main trial who were eligible for the immunology study 39% were excluded due to clinical symptoms at enrolment. The infants enrolled in the immunology study were not different from the general population in the main trial with regard to background characteristics (data not shown). Furthermore, mortality up to three years of age was the same among participants and non-participants in the present immunological study (data not shown).

**Table 1:** Baseline and background characteristics of the study population.

	Males			Females		
	MV	no MV		MV	no MV	
	n (%)	n (%)	p	n (%)	n (%)	p
All	100	73		67	69	
Age (days)	150 (10)	150 (12)	0.91	152 (12)	150 (11)	0.29
Weight (kg)	7.5 (1.0)	7.3 (0.8)	0.17	6.9 (0.7)	6.9 (0.8)	0.85
Length (cm)	65 (3)	64 (2)	0.63	63 (2)	63 (2)	0.65
MUAC (mm)	148 (11)	145 (12)	0.06	142 (11)	145 (11)	0.26
Not breastfed	4 (4%)	3 (4%)	0.97	4 (6%)	3 (4%)	0.67
Extra food supplied	76 (76%)	51 (70%)	0.41	49 (73%)	47 (68%)	0.52
Toilet indoor	84 (84%)	59 (81%)	0.59	52 (78%)	62 (90%)	0.05
Previously hospitalised	2 (2%)	2 (3%)	0.75	2 (3%)	1 (1%)	0.54
OPV vaccinated	100 (100%)	71 (97%)	0.10	65 (97%)	66 (96%)	0.67
BCG vaccinated	99 (99%)	73 (100%)	0.39	67 (100%)	69 (100%)	
VAS 50,000 IU	31 (31%)	22 (30%)		17 (25%)	18 (26%)	
VAS 25,000 IU	26 (26%)	15 (21%)		23 (34%)	18 (26%)	
Placebo (VAS trial)	26 (26%)	20 (27%)		15 (22%)	16 (23%)	
Not in VAS trial	17 (17%)	16 (22%)	0.78*	12 (18%)	17 (25%)	0.68*
Rainy season	71 (71%)	56 (77%)	0.40	49 (73%)	41 (59%)	0.09
In vitro cytokines	81 (81%)	61 (84%)		55 (82%)	53 (77%)	
Plasma cytokines	98 (98%)	72 (99%)		63 (94%)	65 (94%)	
suPAR	96 (96%)	72 (99%)		66 (99%)	63 (91%)	

The percentage is given as the proportion of the reference population. OPV: Oral polio vaccine. MUAC: Mid-upper arm circumference. For the anthropometric measures (age, weight, length and MUAC) the mean (standard deviation) are given. The p-value designating the significance level of the difference between the randomization groups for either sex is obtained by χ2-test for categorical variables, and t-test for continuous variables.

*Statistical test of distribution of all four vitamin A categories.

Baseline levels of plasma biomarkers and *in vitro* cytokines after whole blood stimulation were similar in the intervention and the control groups (data not shown).

### Overall Effect of MV

MV was associated with a higher level of plasma MCP-1, the GMR being 1.11 (1.03–1.19) (Table 2). Other outcome variables were not affected by MV (Table S1 in File S1). Non-parametric analysis of the median corroborated the findings from the regression model of the MV effect (data not shown). For TNF-α in plasma, TNF-α to PHA and LPS, IL-17 to PHA and IL-17 to PPD, singular observations were distinctly low or high relative to the overall distribution of the data, and could hence be regarded as outliers. Omitting these outliers did not substantially affect the estimates of the MV effect (data not shown).

**Table 2:** Effect of MV on plasma biomarkers; overall and stratified by sex.

			All	Males	Females	
	% ND		n = 302	n = 171	n = 131	
	BL	FU	GMR (95% CI)	GMR (95% CI)	GMR (95% CI)	p#
IL-1Ra	0.0	0.0	1.06 (0.96–1.17)	0.95 (0.83–1.09)	1.21 (1.04–1.41)	0.02
MCP-1	0.0	0.0	**1.11 (1.03–1.19)**	1.06 (0.97–1.16)	**1.17 (1.05–1.30)**	0.17
TNF-α	1.3	1.7	1.06 (0.99–1.13)	1.06 (0.96–1.16)	1.06 (0.96–1.18)	0.93
IL-10*	58.3	59.8	1.04 (0.79–1.37)	0.89 (0.64–1.24)	1.35 (0.82–2.22)	0.18
IL-8	0.0	0.0	1.01 (0.91–1.11)	0.92 (0.81–1.05)	1.15 (0.99–1.33)	0.03
IL-6*	88.3	75.2	0.92 (0.61–1.40)	0.92 (0.54–1.57)	0.98 (0.51–1.88)	0.88
suPAR	0.0	0.0	1.00 (0.97–1.03)	0.99 (0.95–1.03)	1.01 (0.97–1.05)	0.52

The percentage of non-detectable measurements (ND, below lower limit of detection) is presented for baseline (BL) and follow-up (FU), respectively. Effect estimates or interactions with a significance level below p = 0.05 after adjustment for multiple testing are highlighted in bold writing.

# P value for interaction between MV and sex.

*Estimates of measles vaccine effect are obtained by Poisson regression due to low number of detectable measurements (<50%).

### Sex-differential Effect of MV

The plasma biomarkers MCP-1 (GMR: 1.17 (1.05–1.30)) and IL-1Ra (GMR: 1.21 (1.04–1.41)) were significantly increased by MV in females, and IL-8 was borderline significantly increased in females (1.15 (0.99–1.33)), with no effect in males. The interaction between sex and MV for IL-1Ra and IL-8 was significant when not adjusting for multiple comparisons (p = 0.02 and p = 0.03, respectively), but lost significance after the adjustment (Table 2). For *in vitro* cytokines, there were no significant effects of MV separately in males and females, and no indications of sex-differential effects of MV (Table S1 in File S1).

### Effect of Symptoms at Follow-up on Effect of MV

Using the same criteria for disease as we did at enrolment, 55% of the infants had symptoms of disease at follow-up (MV: 55%; No MV: 54%, p = 0.88 by χ^2^ test). There was a tendency towards less or even a negative effect of MV on *in vitro* production of inflammatory cytokines in symptomatic infants compared to a generally positive effect of MV in asymptomatic infants (Table S2 in File S1). For the anti-inflammatory IL-10 the opposite trend was found. This effect modification by symptoms was significant for several *in vitro* cytokine outcomes when analysed separately (test for interaction of MV and symptoms: p = 0.002 for TNF-α to LPS; p = 0.02 for IL-17 to PPD; p = 0.01 for IL-17 to TT; p = 0.04 for IL-10 to PHA; p = 0.04 for IL-10 to PPD; p = 0.01 for IL-5 to PPD). However, when adjusting for multiple comparisons, only TNF-α to LPS remained significant (p = 0.05). No significant interaction of symptoms and MV was found for any of the plasma biomarkers (data not shown). The observed effects were similar in the two sexes (data not shown).

### MV and Neonatal Vitamin A Supplementation

The four *ad hoc* sub-groups generated by stratifying by MV and VAS status were not different in respect to the background characteristics (data not shown). Vitamin A supplementation at birth did not significantly modify the effect of MV on plasma biomarkers (data not shown). For *in vitro* cytokine outcomes, MV was associated with an increase in IL-10 to PHA among infants who had not received vitamin A but not in vitamin A recipients (p = 0.03 for interaction of MV and vitamin A). However, after adjustment for multiple comparisons, the interaction between MV and vitamin A was no longer significant (Table S3 in File S1).

There was a trend for vitamin A modifying the effect of MV on the pro- to anti-inflammatory ratio of TNF-α to IL-10 (Table S4 in File S1). In the no-vitamin A group, MV was associated with a reduced TNF-α to IL-10 ratio, whereas MV increased the ratio in vitamin A-recipients. However, after adjustment for multiple comparisons there was no significant interaction between MV and vitamin A.

### MV, Neonatal Vitamin A Supplementation and Sex

The sex-differential effects of MV on IL-1Ra and IL-8 were particularly pronounced in the vitamin A supplemented group (p = 0.01 and p = 0.003 for interaction of MV and sex in vitamin A supplemented infants for plasma IL-1Ra and IL-8, respectively; adjusted for multiple comparisons: p = 0.03 and p = 0.02, respectively), whereas there were no sex-differential effect of MV in infants who did not receive vitamin A (Table 3). There was no interaction between MV and sex on *in vitro* cytokine production when stratifying for vitamin A supplementation (data not shown).

**Table 3:** Effect of MV on plasma biomarkers stratified by vitamin A and sex.

	VAS			No VAS		
	Males	Females		Males	Females	
	n = 94	n = 73		n = 77	n = 58	
	GMR (95% CI)	GMR (95% CI)	p#	GMR (95% CI)	GMR (95% CI)	p#
IL-1Ra	0.89 (0.74–1.08)	**1.35 (1.09–1.69)**	**0.01**	1.05 (0.88–1.27)	1.05 (0.84–1.31)	0.97
MCP-1	1.05 (0.92–1.21)	1.18 (1.01–1.38)	0.27	1.08 (0.95–1.23)	1.17 (1.00–1.36)	0.45
TNF-α	1.04 (0.91–1.18)	1.05 (0.91–1.22)	0.91	1.08 (0.93–1.25)	1.08 (0.90–1.30)	1.00
IL-10*	0.75 (0.47–1.20)	1.31 (0.61–2.79)	0.22	1.04 (0.66–1.65)	1.39 (0.72–2.68)	0.48
IL-8	0.79 (0.65–0.94)	1.21 (0.98–1.50)	**0.003**	1.09 (0.91–1.30)	1.08 (0.87–1.34)	0.95
IL-6*	0.62 (0.30–1.30)	0.97 (0.45–2.10)	0.42	1.54 (0.60–3.98)	0.96 (0.29–3.18)	0.54
suPAR	0.98 (0.93–1.04)	1.04 (0.98–1.10)	0.18	0.99 (0.93–1.05)	0.97 (0.91–1.05)	0.78

VAS: Vitamin A supplementation at birth. Effect estimates or interactions with a significance level below p = 0.05 after adjustment for multiple testing are highlighted in bold writing.

# P value for interaction between MV and sex for vitamin A recipients or non-recipients, respectively.

^*^Estimates of measles vaccine effect are obtained by Poisson regression due to low number of detectables measurements (<50%).

## Discussion

Receiving MV was associated with significantly higher MCP-1 plasma levels, and the effect was significant in its own right in females. Although this did not sustain multiple testing adjustment, indications of sex-differential effects of MV were found for plasma IL-8 and IL-1Ra; MV increased levels in females, but not in males. The *post hoc* stratification by vitamin A indicated that vitamin A amplified the MV effect in females, and that vitamin A shifted the effect of MV in a more pro-inflammatory direction. Additionally, stratification by symptoms at the follow-up visit indicated a differential effect of MV, with a generally positive effect of MV on *in vitro* inflammatory cytokine production in asymptomatic infants.

### Non-specific Effects of MV

The randomised trial setting allowed us to study the immunological effect of MV compared with a control group having DTP3 as the most recent vaccination. The biomarkers affected by MV are associated with immunological defence mechanisms. MCP-1 is a chemokine that attracts a range of leukocyte subsets, particularly phagocytes. MCP-1 plays an important role in commencing the defence against bacterial infections [14]–[16]. IL-8 is a potent chemokine attracting neutrophils to the site of inflammation [17]. IL-1Ra is an antagonist to the IL-1 receptor, and thus acts as a suppressor of the IL-1 pro-inflammatory signalling cascade [18].

Very few studies have assessed the non-specific immunological effects of MV. One study in Bangladeshi 6 months old infants reported that MV was associated with higher lymphocyte expression of the IL-2 receptor α-chain (CD25) on regulatory and activated T-cells, higher expression of the T-cell activation markers CD69 and CD71, a higher frequency of IFN-γ responders, higher levels of IL-2, and a modest increase in IL-10 when measured 6 weeks after MV [7], whereas another study in North American one-year-old children suggested a transient positive effect of MV on non-specific cellular Th1 cytokine responses, although this may have waned 6 weeks after MV [6]. A study in Gambian 3-year-old children found that plasma IL-10, IL-2Rα and MIP-1β had significantly declined two weeks after a booster MV, whereas resting *FOXP3* expression was not affected [19]. Sex interactions were not analysed in any of the two former studies, whereas the latter did not find any sex differences in the biomarkers investigated.

Our results suggest that MV resulted in higher plasma MCP-1 among vaccinated children; however, sex should be taken into account as MV may also increase plasma IL-8 and IL-1Ra in females but not in males. It is still an enigma how the immunomodulation of MV is induced. However, altered immunological responsiveness may be caused by epigenetic reprogramming induced by stimuli, such as vaccines. Epigenetic reprogramming of the innate response pathway has been demonstrated for BCG vaccination [20]. Hence, epigenetic reprogramming may be involved in the non-specific immunological effects of MV.

### Immunomodulation by Vitamin A

Unexpectedly, the main trial of early MV showed that vitamin A interacted with MV. Compared with children who received placebo at birth, neonatal vitamin A was associated with a 2.5-fold higher mortality in MV-vaccinated between 4.5 months and 9 months, strongest so for males [2].

Even though the present study was not designed to examine the effect of vitamin A, the results suggest that MV pushed the TNF-α:IL-10 ratio in a pro-inflammatory direction among children who had received neonatal vitamin A. We have previously proposed that vitamin A amplifies the non-specific effects of vaccines [21], and we have found that vitamin A at birth is associated with increased female mortality after DTP vaccination [22], [23], and vitamin A and DTP vaccination interact on *in vitro* cytokine production in a sex-differential manner [9].

Importantly, our data suggest a long-term immunomodulation by neonatal vitamin A supplementation. The effects were sustained at least up to five months after administration.

### Limitations of the Study

A large number of infants enrolled in the main trial were not eligible for the immunological study due to clinical symptoms. Excluding these children, we may have missed the weakest children, who might benefit the most from MV. However, the mortality in the subgroup included in the current study did not differ from the mortality in the main trial.

Whereas the loss to follow-up was generally low, it was significantly higher among unvaccinated infants than vaccinated infants (10 versus 23, p = 0.01, χ^2^-test). One reason may be that the non-blinded design of the study could have compromised compliance particularly among control infants due to the less immediate benefit of participation in this group.

Samples were drawn 6 weeks after enrolment to minimize the direct immunological response of the MV. The non-specific effects of MV on mortality persist for years [2], [24]. One previous comparable, though much smaller study suggested that *in vitro* IFN-γ, TNF-α, and IL-6 responses to a mitogen decreased transiently shortly after MV, then increased to a second peak at 4 weeks followed by another decline to a level below pre-vaccination at 6 weeks [6]. However, a 6-week follow-up has also been used in a previous study to show immunological effects of MV [7].

Some of the outcomes analysed had a significant proportion of measurements below the detection limit of the assay. This was especially true for IL-6 and IL-10 in plasma and IL-17 after *in vitro* PPD stimulation, where up to 80%, 60% and 50%, respectively were undetectable. The lower sensitivity for these cytokines and subsequent lower resolution of the data may have impaired the possibility to measure an effect of MV.

Analyses were not adjusted for cell subset numbers as leukocyte counts were not performed. The cytokine concentrations may depend on the amount of cells present in the blood sample, which is known to differ between individuals. However, previous studies did not find that MV caused a sustained increase in the number of leukocytes or lymphocytes 6 weeks after vaccination [7], [25], [26].

With its explorative design, the study was not adequately powered to support all the interaction analyses we have performed; some of the results did not sustain after conservative adjustment for multiple comparisons. However, it lends credence to our findings that the effect estimates for MV were all going in the same direction, thus making it less likely that chance mass significance be the mere explanation.

## Conclusion

We found indications that females and males responded immunologically differently to an early measles vaccine at 4.5 month of age, as MV only induced plasma cytokine and receptor changes in females. Previous vitamin A supplementation amplified the pro-inflammatory *in vitro* responses after MV. Albeit the study was explorative and should be interpreted with caution, the study supports the results of epidemiological studies that MV has sex-differential effects on the immune system, and the effect could be modulated by previous vitamin A supplementation.

## Supporting Information

File S1 **Contains the following files.** Table S1: Effect of MV on *in vitro* cytokine production, overall and stratified by sex. Table S2: Effect of MV on *in vitro* cytokine production stratified by presentation of symptoms at follow-up. Table S3: Effect of MV on *in vitro* cytokine production stratified by vitamin A. Table S4: Effect of MV on ratios of *in vitro* cytokine production and plasma biomarker levels stratified by vitamin A
(DOCX)

Checklist S1 CONSORT checklist of the clinical trial.
(DOC)

Protocol S1 **Non-specific effects of vaccines – in search of the immunological background.** The protocol of the immunological study.
(PDF)

## References

[1] Aaby P, Roth A, Ravn H, Napirna BM, Rodrigues A, et al (2011) Randomized trial of BCG vaccination at birth to low-birth-weight children: beneficial nonspecific effects in the neonatal period? J Infect Dis 204: 245–252. 21673035 10.1093/infdis/jir240

[2] Aaby P, Martins CL, Garly ML, Bale C, Andersen A, et al (2010) Non-specific effects of standard measles vaccine at 4.5 and 9 months of age on childhood mortality: randomised controlled trial. BMJ 341: c6495. 21118875 10.1136/bmj.c6495 PMC2994348

[3] Aaby P, Jensen H, Rodrigues A, Garly ML, Benn CS, et al (2004) Divergent female-male mortality ratios associated with different routine vaccinations among female-male twin pairs. IntJEpidemiol 33: 367–373. 10.1093/ije/dyh004 15082642

[4] Aaby P, Benn C, Nielsen J, Lisse IM, Rodrigues A, et al.. (2012) Testing the hypothesis that diphtheria-tetanus-pertussis vaccine has negative non-specific and sex-differential effects on child survival in high-mortality countries. BMJ Open 2. 10.1136/bmjopen-2011-000707 PMC3364456 22619263

[5] Liu L, Johnson HL, Cousens S, Perin J, Scott S, et al (2012) Global, regional, and national causes of child mortality: an updated systematic analysis for 2010 with time trends since 2000. Lancet 379: 2151–2161. 22579125 10.1016/S0140-6736(12)60560-1

[6] Ovsyannikova IG, Reid KC, Jacobson RM, Oberg AL, Klee GG, et al (2003) Cytokine production patterns and antibody response to measles vaccine. Vaccine 21: 3946–3953. 12922130 10.1016/s0264-410x(03)00272-x

[7] Schnorr JJ, Cutts FT, Wheeler JG, Akramuzzaman SM, Alam MS, et al (2001) Immune modulation after measles vaccination of 6–9 months old Bangladeshi infants. Vaccine 19: 1503–1510. 11163674 10.1016/s0264-410x(00)00349-2

[8] Eriksson M, Sartono E, Martins CL, Bale C, Garly ML, et al (2007) A comparison of ex vivo cytokine production in venous and capillary blood. Clin Exp Immunol 150: 469–476. 17924971 10.1111/j.1365-2249.2007.03515.x PMC2219377

[9] Jorgensen MJ, Fisker AB, Sartono E, Andersen A, Erikstrup C, et al.. (2012) The effect of at-birth vitamin A supplementation on differential leucocyte counts and in vitro cytokine production: an immunological study nested within a randomised trial in Guinea-Bissau. Br J Nutr: 1–11. 10.1017/S0007114512001304 23168172

[10] Ostrowski SR, Ullum H, Goka BQ, Hoyer-Hansen G, Obeng-Adjei G, et al (2005) Plasma concentrations of soluble urokinase-type plasminogen activator receptor are increased in patients with malaria and are associated with a poor clinical or a fatal outcome. J Infect Dis 191: 1331–1341. 15776381 10.1086/428854

[11] Uh HW, Hartgers FC, Yazdanbakhsh M, Houwing-Duistermaat JJ (2008) Evaluation of regression methods when immunological measurements are constrained by detection limits. BMC Immunol 9: 59. 18928527 10.1186/1471-2172-9-59 PMC2592244

[12] Andersen A (2012) Statistical Analysis of Population-Based Immunological Studies: Faculty of Health and Medical Sciences, University of Copenhagen.

[13] Newson RB (2010) Frequentist q-values for multiple-test procedures. The Stata Journal 10 (4): 568–584.

[14] Baggiolini M (2001) Chemokines in pathology and medicine. J Intern Med 250: 91–104. 11489059 10.1046/j.1365-2796.2001.00867.x

[15] Chae P, Im M, Gibson F, Jiang Y, Graves DT (2002) Mice lacking monocyte chemoattractant protein 1 have enhanced susceptibility to an interstitial polymicrobial infection due to impaired monocyte recruitment. Infect Immun 70: 3164–3169. 12011011 10.1128/IAI.70.6.3164-3169.2002 PMC127982

[16] Peters W, Dupuis M, Charo IF (2000) A mechanism for the impaired IFN-gamma production in C-C chemokine receptor 2 (CCR2) knockout mice: role of CCR2 in linking the innate and adaptive immune responses. J Immunol 165: 7072–7077. 11120836 10.4049/jimmunol.165.12.7072

[17] Eckmann L, Kagnoff MF, Fierer J (1993) Epithelial cells secrete the chemokine interleukin-8 in response to bacterial entry. Infect Immun 61: 4569–4574. 8406853 10.1128/iai.61.11.4569-4574.1993 PMC281206

[18] Gabay C, Lamacchia C, Palmer G (2010) IL-1 pathways in inflammation and human diseases. Nat Rev Rheumatol 6: 232–241. 20177398 10.1038/nrrheum.2010.4

[19] Njie-Jobe J, Nyamweya S, Miles DJ, van der Sande M, Zaman S, et al (2012) Immunological impact of an additional early measles vaccine in Gambian children: responses to a boost at 3 years. Vaccine 30: 2543–2550. 22314136 10.1016/j.vaccine.2012.01.083 PMC3401374

[20] Kleinnijenhuis J, Quintin J, Preijers F, Joosten LA, Ifrim DC, et al (2012) Bacille Calmette-Guerin induces NOD2-dependent nonspecific protection from reinfection via epigenetic reprogramming of monocytes. Proc Natl Acad Sci U S A 109: 17537–17542. 22988082 10.1073/pnas.1202870109 PMC3491454

[21] Benn CS, Bale C, Sommerfelt H, Friis H, Aaby P (2003) Hypothesis: Vitamin A supplementation and childhood mortality: amplification of the non-specific effects of vaccines? Int J Epidemiol 32: 822–828. 14559758 10.1093/ije/dyg208

[22] Benn CS, Fisker AB, Jorgensen MJ, Aaby P (2008) Conflicting evidence for neonatal vitamin A supplementation. Vaccine 26: 4111–4112. 18486284 10.1016/j.vaccine.2008.04.021

[23] Benn CS, Rodrigues A, Yazdanbakhsh M, Fisker AB, Ravn H, et al (2009) The effect of high-dose vitamin A supplementation administered with BCG vaccine at birth may be modified by subsequent DTP vaccination. Vaccine 27: 2891–2898. 19428899 10.1016/j.vaccine.2009.02.080

[24] Aaby P, Samb B, Simondon F, Seck AM, Knudsen K, et al (1995) Non-specific beneficial effect of measles immunisation: analysis of mortality studies from developing countries. BMJ 311: 481–485. 7647643 10.1136/bmj.311.7003.481 PMC2550544

[25] Rager-Zisman B, Bazarsky E, Skibin A, Tam G, Chamney S, et al (2004) Differential immune responses to primary measles-mumps-rubella vaccination in Israeli children. Clin Diagn Lab Immunol 11: 913–918. 15358652 10.1128/CDLI.11.5.913-918.2004 PMC515267

[26] Snopov SA, Kharit SM, Norval M, Ivanova VV (2005) Circulating leukocyte and cytokine responses to measles and poliovirus vaccination in children after ultraviolet radiation exposures. Arch Virol 150: 1729–1743. 15986177 10.1007/s00705-005-0561-6

