# Supplementary material for: A Randomized Trial of an Early Measles Vaccine at 4½ Months of Age in Guinea-Bissau: Sex-Differential Immunological Effects
Source: PLoS One. 2014 May 16;9(5):e97536. doi: 10.1371/journal.pone.0097536 (PMC4024025; doi:10.1371/journal.pone.0097536)
Supplement: File S1 — Contains the following files. Table S1: Effect of MV on in vitro cytokine production, overall and stratified by sex. Table S2: Effect of MV on in vitro cytokine production stratified by presentation of symptoms at follow-up. Table S3: Effect of MV on in vitro cytokine production stratified by vitamin A. Table S4: Effect of MV on ratios of in vitro cytokine production and plasma biomarker levels stratified by vitamin A (DOCX) [file pone.0097536.s001.docx]

**Supporting Information S1**

**Table S1: Effect of MV on *in vitro* cytokine production, overall and stratified by sex**

|  |  |  |  | **All** | **Males** | **Females** |  |
| --- | --- | --- | --- | --- | --- | --- | --- |
|  |  | % ND | | n=250 | n=142 | n=108 |  |
|  |  | BL | FU | GMR (95% CI) | GMR (95% CI) | GMR (95% CI) | p^#^ |
| TNF-α | PHA | 0.8 | 0.0 | 1.19 (0.89-1.60) | 1.02 (0.69-1.51) | 1.45 (0.93-2.28) | 0.24 |
|  | PPD | 1.6 | 1.6 | 1.21 (0.87-1.69) | 1.19 (0.76-1.85) | 1.27 (0.76-2.13) | 0.85 |
|  | TT | 4.9 | 3.6 | 1.19 (0.81-1.76) | 1.14 (0.68-1.91) | 1.21 (0.67-2.20) | 0.88 |
|  | PAM | 14.9 | 10.8 | 1.25 (0.91-1.71) | 1.25 (0.82-1.92) | 1.18 (0.73-1.91) | 0.85 |
|  | LPS^A^ | 0.0 | 0.4 | 1.02 (0.82-1.27) | 0.88 (0.66-1.18) | 1.20 (0.86-1.69) | 0.17 |
| IL-13 | PHA | 7.2 | 7.2 | 1.17 (0.82-1.65) | 1.02 (0.64-1.62) | 1.36 (0.79-2.32) | 0.43 |
|  | PPD | 42.6 | 48.8 | 1.11 (0.78-1.58) | 1.43 (0.88-2.31) | 0.80 (0.46-1.38) | 0.12 |
|  | TT | 25.5 | 31.6 | 0.94 (0.69-1.27) | 1.00 (0.67-1.50) | 0.82 (0.50-1.34) | 0.54 |
| IL-17 | PHA | 9.2 | 7.2 | 1.05 (0.84-1.31) | 1.17 (0.87-1.58) | 0.90 (0.63-1.27) | 0.25 |
|  | PPD* | 50.2 | 50.4 | 1.00 (0.79-1.27) | 1.24 (0.89-1.73) | 0.77 (0.53-1.12) | 0.06 |
|  | TT | 33.6 | 37.6 | 1.01 (0.73-1.39) | 1.07 (0.71-1.61) | 0.94 (0.58-1.52) | 0.68 |
| IL-10 | PHA | 6.8 | 6.4 | 1.10 (0.81-1.50) | 1.14 (0.75-1.73) | 1.07 (0.66-1.73) | 0.84 |
|  | PPD | 20.5 | 25.2 | 1.10 (0.70-1.72) | 1.15 (0.63-2.11) | 0.95 (0.48-1.89) | 0.69 |
|  | TT | 39.7 | 42.4 | 1.23 (0.71-2.13) | 1.07 (0.52-2.23) | 1.35 (0.61-2.99) | 0.67 |
|  | PAM | 2.8 | 0.8 | 1.00 (0.76-1.33) | 0.93 (0.64-1.35) | 1.08 (0.70-1.66) | 0.60 |
|  | LPS^A^ | 0.0 | 0.4 | 0.99 (0.78-1.26) | 0.87 (0.63-1.20) | 1.15 (0.79-1.67) | 0.26 |
| IFN-γ | PHA | 8.8 | 9.6 | 1.42 (0.90-2.24) | 1.14 (0.63-2.07) | 1.71 (0.85-3.43) | 0.39 |
|  | PPD | 11.2 | 18.8 | 0.96 (0.61-1.51) | 0.93 (0.52-1.68) | 0.98 (0.49-1.99) | 0.90 |
|  | TT | 26.7 | 38.0 | 1.10 (0.66-1.85) | 1.10 (0.55-2.20) | 1.10 (0.48-2.48) | 0.99 |
| IL-5 | PHA | 12.0 | 11.2 | 1.08 (0.66-1.77) | 0.87 (0.45-1.68) | 1.41 (0.66-3.01) | 0.34 |
|  | PPD | 26.1 | 34.4 | 1.25 (0.75-2.08) | 1.17 (0.59-2.33) | 1.39 (0.63-3.07) | 0.75 |
|  | TT | 18.7 | 18.4 | 0.79 (0.50-1.26) | 0.65 (0.36-1.19) | 0.94 (0.47-1.90) | 0.43 |

The proportion of non-detectable measurements (ND, below lower limit of detection) is presented for baseline (BL) and follow-up (FU), respectively.

A: For technical reasons, as <2 observations for these outcomes were below LLD, the estimate was obtained by linear regression with these non-detectable values set to LLD/2.

*) Estimates of measles vaccine effect are obtained by Poisson regression due to low number of detectables measurements (<50%).

#) P value for interaction between MV and sex.

**Table S2: Effect of MV on *in vitro* cytokine production stratified by presentation of symptoms at follow-up**

|  |  | **Ill** | **Not Ill** |  |
| --- | --- | --- | --- | --- |
|  |  | n=129 | n=121 |  |
|  |  | GMR (95% CI) | GMR (95% CI) | p^#^ |
| TNF-α | PHA^A^ | 0.91 (0.60-1.36) | 1.60 (1.05-2.45) | 0.06 |
|  | PPD^A^ | 0.99 (0.62-1.58) | 1.49 (0.92-2.41) | 0.23 |
|  | TT | 1.18 (0.69-2.02) | 1.22 (0.70-2.13) | 0.93 |
|  | PAM | 0.97 (0.63-1.50) | 1.64 (1.04-2.57) | 0.10 |
|  | LPS^A^ | 0.73 (0.54-0.99) | 1.44 (1.06-1.95) | **0.002** |
| IL-13 | PHA | 0.96 (0.59-1.56) | 1.42 (0.86-2.33) | 0.27 |
|  | PPD | 0.83 (0.50-1.38) | 1.40 (0.83-2.36) | 0.17 |
|  | TT | 0.72 (0.47-1.10) | 1.29 (0.82-2.05) | 0.07 |
| IL-17 | PHA | 0.96 (0.70-1.31) | 1.14 (0.82-1.57) | 0.46 |
|  | PPD* | 0.75 (0.53-1.07) | 1.32 (0.95-1.84) | 0.02 |
|  | TT | 0.68 (0.45-1.04) | 1.60 (1.03-2.49) | 0.01 |
| IL-10 | PHA | 1.50 (0.97-2.31) | 0.78 (0.50-1.22) | 0.04 |
|  | PPD | 1.71 (0.92-3.17) | 0.68 (0.37-1.28) | 0.04 |
|  | TT | 2.03 (0.97-4.23) | 0.75 (0.34-1.63) | 0.08 |
|  | PAM | 1.15 (0.78-1.70) | 0.87 (0.58-1.29) | 0.32 |
|  | LPS^A^ | 1.11 (0.79-1.56) | 0.88 (0.62-1.24) | 0.34 |
| IFN-γ | PHA | 1.14 (0.61-2.13) | 1.76 (0.92-3.36) | 0.34 |
|  | PPD | 0.76 (0.41-1.42) | 1.21 (0.64-2.27) | 0.31 |
|  | TT | 0.73 (0.35-1.52) | 1.68 (0.76-3.74) | 0.13 |
| IL-5 | PHA | 0.78 (0.38-1.58) | 1.51 (0.75-3.07) | 0.19 |
|  | PPD | 0.61 (0.30-1.24) | 2.38 (1.15-4.91) | 0.01 |
|  | TT | 0.55 (0.29-1.03) | 1.17 (0.60-2.28) | 0.11 |

Illness is defined by the fulfillment of one or more of the following criteria: current fever or diarrhoea reported by the mother; an axillary temperature above 37.5˚C; a respiratory rate at 60/minute or above; or current infection diagnosed by the physician. Effect estimates or interactions with a significance level below p=0.05 after adjustment for multiple testing are highlighted in bold writing.

A: For technical reasons, as <2 observations for these outcomes were below LLD, the estimate was obtained by linear regression with these non-detectable values set to LLD/2.

*) Estimates of measles vaccine effect are obtained by Poisson regression due to low number of detectables measurements (<50%).

#) P value for interaction between MV and presentation of symptoms at follow-up.

**Table S3: Effect of MV on *in vitro* cytokine production stratified by vitamin A**

|  |  | **VAS** | **No VAS** |  |
| --- | --- | --- | --- | --- |
|  |  | n=143 | n=107 |  |
|  |  | GMR (CI 95%) | GMR (CI 95%) | p^#^ |
| TNF-α | PHA | 1.29 (0.87-1.91) | 1.05 (0.67-1.64) | 0.50 |
|  | PPD | 1.33 (0.85-2.06) | 1.09 (0.66-1.82) | 0.57 |
|  | TT | 1.31 (0.78-2.21) | 1.10 (0.61-1.99) | 0.66 |
|  | PAM | 1.37 (0.90-2.09) | 1.10 (0.68-1.79) | 0.50 |
|  | LPS^A^ | 1.13 (0.85-1.51) | 0.93 (0.67-1.29) | 0.37 |
| IL-13 | PHA | 1.06 (0.67-1.67) | 1.18 (0.70-2.00) | 0.75 |
|  | PPD | 0.90 (0.57-1.42) | 1.32 (0.73-2.38) | 0.31 |
|  | TT | 0.96 (0.63-1.47) | 0.89 (0.55-1.45) | 0.81 |
| IL-17 | PHA | 1.01 (0.75-1.35) | 1.05 (0.75-1.47) | 0.87 |
|  | PPD* | 0.95 (0.70-1.28) | 1.04 (0.71-1.53) | 0.71 |
|  | TT | 1.10 (0.74-1.65) | 0.88 (0.54-1.45) | 0.48 |
| IL-10 | PHA | 0.81 (0.54-1.23) | 1.60 (1.00-2.57) | 0.03 |
|  | PPD | 1.03 (0.57-1.85) | 1.27 (0.64-2.52) | 0.64 |
|  | TT | 1.21 (0.59-2.51) | 1.38 (0.61-3.12) | 0.81 |
|  | PAM | 0.91 (0.62-1.32) | 1.09 (0.72-1.67) | 0.52 |
|  | LPS^A^ | 0.98 (0.71-1.36) | 1.05 (0.73-1.51) | 0.80 |
| IFN-γ | PHA | 1.39 (0.76-2.55) | 1.34 (0.67-2.67) | 0.94 |
|  | PPD | 1.14 (0.61-2.10) | 0.68 (0.34-1.35) | 0.27 |
|  | TT | 1.39 (0.67-2.88) | 0.88 (0.37-2.08) | 0.44 |
| IL-5 | PHA | 1.05 (0.54-2.01) | 1.03 (0.49-2.16) | 0.97 |
|  | PPD | 1.23 (0.63-2.42) | 1.16 (0.54-2.49) | 0.91 |
|  | TT | 1.07 (0.59-1.96) | 0.54 (0.27-1.07) | 0.14 |

Effect estimates or interactions with a significance level below p=0.05 are highlighted in bold writing. VAS: Vitamin A supplementation at birth.

A: For technical reasons, as <2 observations for these outcomes were below LLD, the estimate was obtained by linear regression with these non-detectable values set to LLD/2.

*) Estimates of measles vaccine effect are obtained by Poisson regression due to low number of detectables measurements (<50%).

#) P value for interaction between MV and VAS.

**Table S4: Effect of MV on ratios of *in vitro* cytokine production and plasma biomarker levels stratified by vitamin A**

|  |  | **VAS** | **No VAS** |  |
| --- | --- | --- | --- | --- |
|  |  | GMRR (95% CI) | GMRR (95% CI) | p^#^ |
| TNF-α:IL-10 | PHA | 1.58 (0.97-2.59) | 0.67 (0.38-1.17) | 0.02 |
|  | PPD | 1.29 (0.76-2.20) | 0.87 (0.47-1.59) | 0.33 |
|  | LPS^A^ | 1.15 (0.76-1.75) | 0.88 (0.55-1.42) | 0.40 |
|  | plasma | 1.20 (0.79-1.82) | 0.84 (0.56-1.27) | 0.22 |

Effect estimates or interactions with a significance level below p=0.05 are highlighted in bold writing. VAS: Vitamin A supplementation at birth.

A: For technical reasons, as <2 observations for these outcomes were below LLD, the estimate was obtained by linear regression with these non-detectable values set to LLD/2.

#) P value for interaction between MV and VAS.
