# Supplementary material for: A Randomized Trial of an Early Measles Vaccine at 4½ Months of Age in Guinea-Bissau: Sex-Differential Immunological Effects
Source: PLoS One. 2014 May 16;9(5):e97536. doi: 10.1371/journal.pone.0097536 (PMC4024025; doi:10.1371/journal.pone.0097536)
Supplement: Protocol S1 — Non-specific effects of vaccines – in search of the immunological background. The protocol of the immunological study. (PDF) [file pone.0097536.s003.pdf]

NON-SPECIFIC EFFECTS OF VACCINES
# - IN SEARCH OF THE IMMUNOLOGICAL BACKGROUND -

OBJECTIVES

- General: To investigate the immunological background for the non-specific effects of diphtheria-tetanus-pertussis (DTP) and measles vaccines on child mortality
- Specific: Examine the cytokine responses and possible association with morbidity in a study of DTP vaccinated children who will be randomised to receive a measles vaccine or no vaccine at 4½ months of age. (All children will receive a measles vaccine at 9 months of age).

INTRODUCTION / BACKGROUND

A multitude of epidemiological analyses on vaccination data suggest that basically all the vaccines used in the Expanded Programme on Immunization (EPI) are associated with non-specific effects on child mortality in countries with high child mortality. Bacillus Calmette Guerin (BCG), measles and oral polio (OPV) vaccines are associated with decreased child mortality, while the opposite seems to be the case for DTP and hepatitis B (HBV) vaccines. The background for this phenomenon is virtually unknown, but has been ascribed to stimulation by the vaccine of the immune system rendering the individual either more resistant or more prone to succumbing to the high infectious pressure in these environments.

BACKGROUND

Non-specific effects of vaccines

The idea of vaccines having non-specific effects was first proposed in 1991 from a study in Senegal, West Africa. It was discovered that children receiving high-titre measles vaccine (HT) at 6 months of age had higher mortality than children who received the standard titre measles vaccine (STD) at 9 months of age [1 2 3]. The difference was found only for girls. A study from Haiti confirmed the effect [4]. Since children vaccinated with HT had lower mortality than their equivalents who had not received any measles vaccine, the difference in mortality between recipients of HT vaccine and STD vaccine was explained by a non-specific beneficial effect of the STD measles vaccine rather than a harmful effect of the HT vaccine [5 6]. The non-specific beneficial effect of STD measles vaccine on child mortality has been reconfirmed in many data-sets [7 8 9 10 11].

Also the BCG vaccine is associated with striking effects on child mortality reducing mortality by about 50% [12]. Further, among BCG vaccinated children, having a BCG scar or a positive tuberculin reaction was associated with about 55% lower mortality in the following 12 months than among children who had a negative tuberculin reaction or who did not have a BCG scar [13 14].

The effect of OPV is difficult to separate from the effects of BCG and DTP vaccines since OPV is normally given together with these vaccines. There have, though, been some periods without DTP in Bissau due to global shortage of vaccines, and we have compared the case fatality at the hospital for children who received only OPV and children who received both the prescribed OPV and DTP. Children having received OPV had 3-fold lower mortality than children having received both vaccines [15]. Data from an OPV vaccination campaign that took place in Guinea-Bissau also suggested a non-specific beneficial effect for the recipients [16]. Further, studies from Chile and the Soviet Union have suggested that OPV had a beneficial effect on mortality and morbidity [17].

In contrast, DTP, HBV and inactivated polio vaccine (IPV) seem to exert a non-specific detrimental effect on child mortality [12 18 19 20 21 22 23], although the findings on DTP were considered controversial by a recent review [24]. Current studies indicate that the negative effect of

DTP may be neutralized by a subsequent measles vaccination [25 26]. It is striking that all the vaccines with a non-specific beneficial effect are live, whereas the vaccines with an apparently harmful effect are killed. Results from animal studies have shown that attenuated live vaccines tend to induce a Th1 response and offer better protection against severe disease than the corresponding inactivated vaccines, which tend to induce a Th2 response [27 28 29 30]. So far, very few studies have examined whether these effects differ between male and female animals. One study reported that BCG-vaccinated female mice were better protected against malaria parasites than male mice [31]. There is therefore an urgent need to conduct studies that can help uncover the immunology behind the non-specific effects.

Sex-specific effects

All epidemiological studies carried out so far confirms the observation that non-specific effects are sex-specific [32]. Live vaccines (measles, BCG, OPV) have a beneficial effect that is particularly good for girls whereas inactivated vaccines (DTP, HBV, IPV) have a negative effect for girls. To date, there are no immunological studies which have examined whether routine vaccines affect the immune system differently for boys and girls.

We thus propose to study, in a randomised controlled trial of measles vaccination taking place in Guinea-Bissau, the immunology of non-specific effects of vaccination, and their interaction with sex. Specifically, among children who have received the 3 recommended doses of DTP, we will be able to compare the cytokine and antibody profiles of children who receive an early dose of measles vaccine at 4½ months of age with children who receive no additional vaccine at this age (please refer to methods).

METHODS

Study site

The Bandim Health Project (BHP) has a demographic surveillance system in several districts of the capital of Guinea-Bissau, and the current population in these districts is around 80.000. All houses in the area are visited every month to register new pregnancies and births. The BHP visits all children at home every 3 months until the age of three years. Information is collected routinely on breastfeeding patterns, infections, hospitalisation, vaccination status, living with the mother, and ownership of pigs. The people of Bissau are travelling a lot due to extended families living in other parts of the country, cashew harvest, and trade. About 20% of the mothers are travelling at any given time. About 2.600 children are born in the BHP study area each year, and experience shows that we will be able to enrol about 80% of these in a measles vaccine trial i.e. about 2.000 infants per year.

Measles vaccination trial

We are currently conducting a randomised measles vaccination trial with the following schedule:

	Very early two-dose	9 months one- or two-dose	
	st-EZ	st-SW or	st-EZ
	**arm I**	**arm II**	**arm III**
4½ mo. of age	V ¤		
9 mo. of age	V ¤	V ¤	V ¤
18 mo. of age		V/nothing ¤	V/nothing ¤
24 mo. of age	¤	¤	¤

V = measles vaccination, ¤ = Blood sampling, st-EZ = standard-titre Edmonston-Zagreb measles vaccine, st-SW = standard titre Schwarz measles vaccine

The aims of the trial are to examine:

- whether the negative effect of the DTP vaccine, which is administered by the EPI-programme at 6, 10 and 14 weeks of age, can be reverted by administration of a measles vaccine 4 weeks after the last DTP vaccination at around 4½ months of age
- whether the standard-titre Edmonston-Zagreb (EZ) vaccine will be suitable for use in a very early two-dose schedule vaccinating at 4½ and 9 months of age, and
- whether the standard titre Schwarz (SW) or standard-titre Edmonston-Zagreb (EZ) measles vaccine will be the best vaccine strain for use in a routine one-dose measles vaccination schedule and a two-dose measles vaccination schedule in terms of antibody response, protection against measles and child survival

Preliminary results, measles vaccination trial

The study was initiated in August 2003, and has currently recruited 2025 children. All routines are working well. A morbidity survey was implemented in August 2004.

It is too early to say anything about relative mortality in the three study arms. However, BHP has for many years registered all paediatric hospitalisations in Bissau which allows us to use hospitalisations as a morbidity outcome. Preliminary data on hospitalisations from the paediatric department in Bissau suggest that as predicted early measles vaccination protects strongly against hospitalisation for measles infection (see Table) but may also be beneficial, at least for girls, in protecting against hospitalisations for other causes. These preliminary results from a randomised trial corroborate our previous observational studies showing measles vaccine to be associated with a low female-male mortality ratio and DTP with a higher female-male ratio.

**Hospitalisations (cases/children at risk) between 1st and 2nd vaccine in two-dose trial**			
	**EZ measles vaccine at 4½ months**	**No measles vaccine at 4½ months**	**RR (EZ / no measles vaccine)**
**Hospitalisations for measles infection**			
**Male**	0 (0/333)	0.01 (10/698)	0 (0-0.94)
**Female**	0 (0/344)	0.01 (6/647)	0 (0-1.60)
**All**	0 (0/677)	0.01 (16/1345)	0 (0-0.52)
**Hospitalisation for non-measles infections**			
**Male**	0.02 (8/333)	0.03 (20/698)	0.84 (0.32-1.99)
**Female**	0.01 (5/344)	0.04 (29/647)	0.32 (0.10-0.85)
**All**	0.02 (13/677)	0.04 (49/1345)	0.53 (0.26-0.99)

The proposed study will be conducted as an immunological study performed among a subgroup of the participants in the above study.

Procedures, measles vaccination trial

Newborn infants are identified in the BHP registration system. To make sure that all children have received three doses of DTP before inclusion in the Two-dose trial, we contact the mothers of 6, 10 and 14 weeks old children, and remind them to go to the local health centre to receive the DTP

vaccine. The formal inclusion in the Two-dose trial is at 4½ months of age. During the morning hours field-workers contact the mothers/guardians of the children to be included in the study. The field-workers explain about the study, perform a questionnaire, and obtain verbal consent (please refer to ethical considerations).

In the afternoon, the mothers/guardians of children present at the local health centre with the baby to receive oral (in Portuguese Creole) and written (in Portuguese) explanation about the study from the physician and to give consent for participation. The children belonging to arm I of the study will receive vaccination with the standard-titre EZ vaccine. At each visit to the health centre a physician will perform a medical examination. If the child is so ill that it needs to be hospitalised, it will only be able to participate in the study after it has recovered.

Additional procedures, proposed immunological study

In the proposed study it is planned to perform the first blood sample on the first contact at the health centre at 4½ months of age. At this age, all the children have received the third dose of DTP. On the same occasion, participants in arm I will receive a measles vaccine. Six weeks after this occasion (at 6 months of age), a blood sampled will be obtained at home. Children enrolled in the immunological study will be included in a morbidity study to be able to assess the possible correlation between differences in cytokine profile and morbidity.

Immunological analyses

The project provides a unique opportunity to perform detailed immunological analyses in a vaccination trial in a resource-poor developing country in a manner that will not be technically demanding nor taxing for the study population. Considering the limitation of only measuring a Th1 marker (IFN-g) in the face of growing evidence that a balance not only between Th1 and Th2 responses but also pro and anti-inflammatory networks are crucial for beneficial outcome of diseases and most probably vaccines, we will also measure IL-5, IL-13 as Th2, IL-10 as anti-inflammatory and TNF-a and IL-1 as pro inflammatory markers. With the advent of the multiplexed particle-based flow cytometric assays for simultaneous measurements of cytokines or antibodies in small volumes of biological samples it is now possible to conduct immuno-epidemiological studies without compromising the population sample size [33]. This assay has been optimised for use in our setting, and is currently being used in Bissau in collaboration with Leiden University Medical Centre, The Netherlands. All blood samples will be collected by finger-prick in microtubes. Each participant to be blood sampled will have a blood smear prepared and examined for malaria parasitaemia. Participants with malaria will be treated according to local recommendations.

Whole blood culture

The blood will be diluted 1:10 with RPME-1640 medium and cultured in 96 well round bottomed plates. Stimulations will besides from control medium be performed with mitogens: PHA, anti-CD3+anti-CD28, and LPS. PHA is a strong stimulus for T cell derived cytokines particularly for IFN-g, IL-13 and IL-5. Anti CD3+CD28 is a strong stimulus for stimulating IL-10 production from T cells whereas LPS will provide us with information on responsiveness of the innate immune system mediated via Toll Like Receptor 4. Furthermore, the following antigens will be used: PPD and TT. Supernatants will be collected on days 1 and 3 and frozen at minus 80 degrees until use.

Cytokine determinations

The representative cytokines of the Th1, Th2 and regulatory T-cells will be measured simultaneously in the multiplexed particle-based flow cytometric assays. This newly developed assay system which in theory would allow the detection of more than 50 different cytokines in a

small volume of 20 ul, is now operational at a level which allows the determination of now up to 8 cytokines simultaneously. The assay employs beads that are dyed with two fluorophores at varying ratios; different ratios therefore distinguish the test bead sets. Specific capture antibodies are coupled to specific bead sets and the different bead sets can be mixed together which means different cytokine capture antibodies can be added to the same sample. This refinement on the cytokine detection technology is of particular importance to immuno-epidemiological studies where volume of blood is a serious limiting factor.

Antibody determinations
Measles antibodies will be analysed at the Medical Research Laboratories in The Gambia by haemagglutination inhibition test (HAI), which has been in routine use for many years in The Gambia [34] [35].

Morbidity survey
The children enrolled in the immunological study will be followed with morbidity visits between 4½ and 9 months of age to assess disease episodes, consultations and vaccine adverse events. The morbidity survey collects information on diarrhoea, upper and lower respiratory tract infections, abnormalities of the skin, the general well-being of the child including measurement of temperature, consultations and hospitalisations. Within the first two weeks after vaccination the child will be visited 5 times, thereafter once per week. In case of diarrhoea, a faecal specimen will be collected [36] and analysed for parasites and rotavirus. In case of fever (an axillary temperature $\geq 37.5^{o}C$), a blood smear will be collected for determination of malaria parasitaemia. In case of lower respiratory infection or other signs of severe disease, the mother will be urged to consult at the local health centre.

Further, the BHP monitors consultations at the local health centres and hospitalisations at the one paediatric department in Bissau. From these registers additional information on the children will be added.

HIV-infection in Bissau
The population in the study area has a high prevalence of HIV-2 infection (6-9%). However, very few children get HIV-2 infected and HIV-2 is therefore not expected to be an important confounder in the proposed studies. The prevalence of HIV-1 was 2.5% in the last survey before the war and is probably around 3-4 % at the moment among women of fertile age. Since the epidemic is recent, most mothers will be in the early phase of infection and vertical transmission is therefore expected to be somewhat lower than in other regions, presumably around 25%. We may ask for permission to carry out anonymous testing of blood samples that might be available from children who died to assess whether there is any indication of a differential effect for HIV-infected and uninfected children. Such permission has been granted in previous studies in Bissau.

Sample size
Morbidity
With an expected diarrhea frequency of 6 episodes per year for children under 3 years (36), we can assume that each child will have at least one episode of diarrhea or fever between 4 ½ and 9 months of age. With 200 vaccinated and 200 unvaccinated children followed for morbidity, we will with a power of 80% be able to find a 20% reduction in infection incidence.
Preliminary results indicate a 50% reduction in hospitalization rates for measles vaccination at 4 ½ months (table). Not only those who have taken the blood test, but all children (n = 5,755) included in the study will be registered in terms of hospitalizations. One third of the children enrolled at 4 ½

months will receive measles vaccine immediately, and 2/3 will receive measles vaccine at 9 months of age. With 765 vaccinated at 4 ½ months and 1,530 unvaccinated (who will receive the vaccine at 9 months) we will be able to find a 50% reduction in hospitalization with 80% power.

Immunology
Preliminary studies have shown a baseline level of IFN-g at 69 pg / ml. With 200 vaccinated and 200 controls we will be able to detect a 25% increase in IFN-g, and a 25% increase in IL-10 from 18 to 24 pg / ml.

Time table
The study is planned to begin in January 2005 with a recruitment period of about 8 months. Since season has a large influence on health in West Africa on both morbidity [37] and mortality [38], as well as immunologic parameters such as delayed-type hypersensitivity, T-cells, thymus size and measles antibody levels [13 39 40 41], it will be desirable to spread out the sample collection to cover both seasons of the year. Thus, we plan to include 25 children to be measles vaccinated per month, and 25 controls alternating from arm II and arm III.

Analysis
The analysis will emphasise changes in immunological parameters between the first and second sample for the two groups to assess the effect of a measles vaccine after DTP controlling for age related changes. Possible differences between boys and girls will be examined. This analysis is essentially an explorative investigation to detect immunological changes which might explain the differential effects of DTP and measles vaccine. At the same time, it will be investigated whether these potential differences in cytokine profile correlate with differences in morbidity between 4½ and 9 months of age when all children receive measles vaccine.

QUALIFICATIONS OF THE APPLICANT

The applicant has been employed as a medical researcher at the Statens Serum Institut, Denmark, and the Bandim Health Project, Guinea-Bissau, since 1994, and has completed a PhD thesis and several papers on specific and non-specific effects of measles and BCG vaccination [42 43 44 13 45 14 22]. Thus, the applicant has a sound basis for further research into these areas, and a great interest in continued research efforts that could lead to strengthening of the local Guinean research milieu, and hopefully leading to improved child health.

PUBLICATION OF FINDINGS

The analyses of data and the findings from the study will be discussed in the group of academics working on the project. The results will be made available to the Ministry of Health and health professionals in Guinea-Bissau, and we plan to publish the results in peer reviewed international journals.

ETHICAL CONSIDERATIONS

The two-dose study of measles vaccination was approved by the Ministry of Health in Guinea-Bissau, the MRC Ethical Committee and the Scientific Coordinating Committee at the MRC in The Gambia, and by the Danish Central Ethical Committee. The present protocol will be submitted to the same committees.

Mothers/guardians will be informed about the measles vaccination trial shortly before the child reaches 4½ months of age (see Procedures, measles vaccination trial). Since most mothers are illiterate there is no tradition for written consent in the study area. However, mothers who feel

confident will be asked to sign, and those who cannot write will be asked to confirm their participation by fingerprint. Mothers accepting to participate will be asked to draw a lot to emphasise that the study is a randomised trial. The mothers will be asked to consent to participate after having received an explanation, which will contain the following information verbally (Portuguese Creole) and in print (Portuguese):

Normally children receive one measles vaccine at 9 months of age. Since many children below 9 months of age risk to get measles we would like to find out whether it is best to get two doses of measles vaccine at 4½ and 9 months of age, or one dose at 9 months of age, or two doses at 9 and 18 months of age. We would like to test which of two types of measles vaccines will be the best for these schedules. Your child will either receive two doses at 4½ and 9 months of age, one dose at 9 months of age, or two doses of vaccine at 9 and 18 months of age. When your child is 4½ months of age you will draw a lottery, and if the child receives the first vaccination at 9 months of age, you will draw a lottery again at 18 months of age.

Arm I: Your child has been selected by lottery to participate in the part of the study where your child will receive the first dose of vaccine at 4½ months of age, and the second dose at 9 months of age. We will take a blood sample by finger-prick from your child the first time he is vaccinated. Six weeks later when your child is 6 months of age, we will visit you at home to draw a second blood sample. We will take a third blood sample the second time your child is vaccinated at 9 months of age, and a fourth sample at 2 years of age to test whether he is protected against measles ("strength in the blood").

Arm II+III: Your child has been selected by lottery to participate in the part of the study where your child will receive the first vaccine at 9 months of age. At 18 months of age, you will draw a lot again to decide whether he should receive a second dose of measles vaccine or not. We will take a blood sample by finger-prick from your child at 4½ months of age at the first visit to the health centre. Six weeks later when your child is 6 months of age, we will visit you at home to draw a second blood sample. We will take a third blood sample the second time you visit the health centre at 18 months of age, and a fourth sample at 24 months of age to test whether he is protected against measles ("strength in the blood").

All arms: When we take blood samples, we will test whether your child has malaria, and treat him if necessary. At all visits to the health centre he will be clinically examined by a physician, and treated if necessary. You will not have to pay for this treatment. The results of these studies may contribute to better vaccination policies in the future. It is voluntary to participate, and you can withdraw from the study at any time. Do you have any questions about the study?

STUDY GROUP

May-Lill Garly MD PhD DTM&H
Cesário L. Martins MD MSc PhD-Student
Amabelia Rodrigues PhD
Maria Yazdanbakhsh PhD
Hilton C. Whittle MD FRCP
Ida M. Lisse MD
Christine Stabell Benn MD PhD
Peter Aaby MSc DMSc

Reference List

(1) Aaby P, Samb B, Simondon F, Whittle HC, Seck AM, Knudsen K et al. Child mortality after high-titre measles vaccines in Senegal: the complete data set. Lancet 1991; 338:1518-1519.
Ref ID: 62

(2) Garenne M, Leroy O, Beau J-P, Sene I. Child mortality after high-titre measles vaccines: prospective study in Senegal. Lancet 1991; 338(Oct. 12):903-907.
Ref ID: 524

(3) Aaby P, Knudsen K, Whittle HC, Lisse IM, Thaarup J, Poulsen A et al. Long-term survival after Edmonston-Zagreb measles vaccination in Guinea-Bissau: Increased female mortality rate. Pediatrics 1993; 122(8):904-908.
Ref ID: 737

(4) Holt EA, Moulton LH, Siberry GK, Halsey NA. Differential mortality by measles vaccine titer and sex. J Infect Dis 1993; 168:1087-1096.
Ref ID: 310

(5) Aaby P, Samb B, Simondon F, Knudsen K, Seck AM, Bennett J et al. A comparison of vaccine efficacy and mortality during routine use of high-titre Edmonston-Zagreb and Schwarz standard measles vaccines in rural Senegal. Trans R Soc Trop Med Hyg 1996; 90(3):326-330.
Ref ID: 1823

(6) Aaby P, Samb B, Simondon F, Knudsen K, Seck AM, Bennett J et al. Five year follow-up of morbidity and mortality among recipients of high- titre measles vaccines in Senegal. Vaccine 1996; 14(3):226-229.
Ref ID: 1816

(7) Aaby P, Andersen M, Sodemann M, Jakobsen M, Gomes J, Fernandes M. Reduced childhood mortality after standard measles vaccination at 4-8 months compared with 9-11 months of age. BMJ 1993; 307:1308-1311.
Ref ID: 18

(8) Aaby P, Samb B, Simondon F, Seck AMC, Knudsen K, Whittle HC. Non-specific beneficial effect of measles immunisation: analysis of mortality studies from developing countries. BMJ 1995; 311:481-485.
Ref ID: 295

(9) Desgrées du Loû A, Pison G, Aaby P. Role of immunizations in the recent decline in childhood mortality and the changes in the female/male mortality ratio in rural Senegal. Am J Epidemiol 1995; 142(6):643-652.
Ref ID: 832

(10) Knudsen KM, Aaby P, Whittle H, Rowe M, Samb B, Simondon F et al. Child mortality following standard, medium or high titre measles immunization in West Africa. Int J

Epidemiol 1996; 25(3):665-673.
Ref ID: 1817

(11) Aaby P, Garly M-L, Bale C, Martins C, Jensen H, Lisse I et al. Survival of previously measles-vaccinated and measles-unvaccinated children in an emergency situation: an unplanned study. Pediatr Infect Dis J 2003; 22(9):798-805.
Ref ID: 1426

(12) Kristensen I, Aaby P, Jensen H. Routine vaccinations and child survival: follow up study in Guinea- Bissau, West Africa. BMJ 2000; 321(7274):1435-1438.
Ref ID: 782

(13) Garly M-L, Bale C, Martins CL, Balde MA, Hedegaard KL, Whittle HC et al. BCG vaccination among West African infants is associated with less anergy to tuberculin and diphtheria-tetanus antigens. Vaccine 2001; 20(3-4):468-474.
Ref ID: 887

(14) Garly M-L, Martins CL, Bale C, Balde MA, Hedegaard KL, Gustafson P et al. BCG scar and positive tuberculin reaction associated with reduced child mortality in West Africa. A non-specific beneficial effect of BCG? Vaccine 2003; 21(21-22):2782-2790.
Ref ID: 1427

(15) Aaby P, Rodrigues A, Biai S, Martins C, Veirum JE, Benn CS et al. Oral polio vaccination and low case fatality at the paediatric ward i Bissau, Guinea-Bissau. Vaccine 2004; in press.
Ref ID: 2360

(16) Aaby P, Sodemann M, Nhante E, Veirum JE, Jakobsen M, Lisse IM et al. Childhood mortality after a national polio immunization: A beneficial effect? Draft 2000.
Ref ID: 1489

(17) Voroshilova MK. Potential use of nonpathogenic enteroviruses for control of human disease. Prog Med Virol 1989; 36:191-202.
Ref ID: 2324

(18) Veirum JE, Sodemann M, Biai S, Jakobsen M, Garly M-L, Hedegaard KL et al. Routine vaccinations associated with divergent effects on female and male mortality at the paediatric ward in Bissau, Guinea-Bissau . Vaccine 2004; In press.
Ref ID: 1991

(19) Aaby P, Jensen H, Samb B, Cisse B, Sodemann M, Jakobsen M et al. Differences in female-male mortality after high-titre measles vaccine and association with subsequent vaccination with diphtheria-tetanus-pertussis and inactivated poliovirus: reanalysis of West African studies. Lancet 2003; 361(9376):2183-2188.
Ref ID: 1848

(20) Aaby P, Jensen H, Simondon F, Whittle H. High-titer measles vaccination before 9 months of age and increased female mortality: do we have an explanation? Semin Pediatr Infect Dis 2003; 14(3):220-232.
Ref ID: 2259

(21) Aaby P, Jensen H, Gomes J, Fernandes M, Lisse IM. The introduction of diphtheria-tetanus-pertussis vaccine and child mortality in rural Guinea-Bissau: an observational study. Int J Epidemiol 2004; 33(2):374-380.
Ref ID: 1428

(22) Garly M-L, Jensen H, Martins CL, Bale C, Baldé MA, Lisse IM et al. Hepatitis-B vaccination associated with an increased female–male mortality ratio in Guinea-Bissau: an observational study. Pediatr Infect Dis J 2004; in press.
Ref ID: 1488

(23) Aaby P, Garly M-L, Jensen H, Martins C, Bale C, Benn CS et al. Is inactivated polio vaccination associated with increased female mortality? Community studies from Guinea-Bissau. Int J Epidemiol 2004; submitted.
Ref ID: 1792

(24) Evans S, Hall A, Hussey GD, Lanata CF, Radhakrishna S, Smith P et al. WHO Task Force on Routine Infant Vaccination and Child Survival: Report of a meeting to review evidence for a deleterious effect of DPT vaccination on child survival. 2004.
Ref Type: Report
Ref ID: 2672

(25) Aaby P, Jensen H, Garly M-L, Bale C, Martins C, Lisse I. Routine vaccinations and child survival in a war situation with high mortality: effect of gender. Vaccine 2002; 21(1-2):15-20.
Ref ID: 1482

(26) Veirum JE, Sodemann M, Biai S, Jakobsen M, Garly M-L, Hedegaard KL et al. Diphtheria-tetanus-pertussis and measles vaccinations associated with divergent effects on female and male mortality at the paediatric ward in Bissau, Guinea-Bissau. submitted 2002.
Ref ID: 1991

(27) Lindblad EB, Elhay MJ, Silva R, Appelberg R, Andersen P. Adjuvant modulation of immune responses to tuberculosis subunit vaccines. Infect Immun 1997; 65(2):623-629.
Ref ID: 1438

(28) Fischer JE, Johnson JE, Johnson TR, Graham BS. Pertussis toxin sensitization alters the pathogenesis of subsequent respiratory syncytial virus infection. J Infect Dis 2000; 182(4):1029-1038.
Ref ID: 1592

(29) Huber SA, Pfaeffle B. Differential Th1 and Th2 cell responses in male and female BALB/c mice infected with coxsackievirus group B type 3. J Virol 1994; 68(8):5126-5132.
Ref ID: 1480

(30) Pittman PR. Aluminum-containing vaccine associated adverse events: role of route of administration and gender. Vaccine 2002; 20 Suppl 3:S48-S50.
Ref ID: 1944

(31) Clark IA, Allison AC, Cox FE. Protection of mice against Babesia and Plasmodium with BCG. Nature 1976; 259(5541):309-311.
Ref ID: 1590

(32) Aaby P, Samb B, Simondon F, Knudsen K, Seck AMC, Bennett J et al. Sex-specific differences in mortality after high-titre measles immunization in rural Senegal. Bull World Health Organ 1994; 72(5):761-770.
Ref ID: 240

(33) Vignali DA. Multiplexed particle-based flow cytometric assays. J Immunol Methods 2000; 243(1-2):243-255.
Ref ID: 2620

(34) Whittle HC, Mann G, Eccles M. Effects of dose and strain of vaccine on success of measles vaccination of infants aged 4-5 months. Lancet 1988; i:963-966.
Ref ID: 268

(35) Whittle HC, Campbell H, Rahman S, Armstrong JRM. Antibody persistence in Gambian children after high-dose Edmonston-Zagreb measles vaccine. Lancet 1990; 336:1046-1048.
Ref ID: 277

(36) Valentiner-Branth P, Steinsland H, Fischer TK, Perch M, Scheutz F, Dias F et al. Cohort Study of Guinean Children: Incidence, Pathogenicity, Conferred Protection, and Attributable Risk for Enteropathogens during the First 2 Years of Life. J Clin Microbiol 2003; 41(9):4238-4245.
Ref ID: 2276

(37) Molbak K, Jensen H, Ingholt L, Aaby P. Risk Factors for Diarrheal Disease Incidence in Early Childhood: A Community Cohort Study from Guinea-Bissau. Am J Epidemiol 1997; 146(3):273-282.
Ref ID: 528

(38) Moore SE, Cole TJ, Poskitt EM, Sonko BJ, Whitehead RG, McGregor IA et al. Season of birth predicts mortality in rural Gambia [letter]. Nature 1997; 388(6641):434.
Ref ID: 1256

(39) Lisse IM, Aaby P, Whittle HC, Jensen H, Engelmann MDM, Christensen LB. T-lymphocyte subsets in West African children: Impact of age, sex, and season. J Pediatr 1997; 130(1):77-85.
Ref ID: 426

(40) Aaby P, Marx C, Trautner SL, Rudå D, Hasselbalch H, Jensen H et al. Thymus size at birth is associated with infant mortality: a community study from Guinea-Bissau. Acta Paediatr 2002; 91:698-703.
Ref ID: 1465

(41) Whittle HC, Aaby P, Samb B, Cisse B, Kanteh F, Soumaré M et al. Poor serological responses 5-7 years after immunization with high and standard titre measles vaccines.

Pediatr Infect Dis J 1999; 18(1):53-57.
Ref ID: 577

(42) Garly M-L, Martins CL, Balé C, da Costa F, Dias F, Whittle HC et al. Early two-dose measles vaccination schedule in Guinea-Bissau: Good protection and coverage in infancy. Int J Epidemiol 1999; 28(2):347-352.
Ref ID: 802

(43) Garly M-L. Specific and non-specific effects of the standard-titre measles vaccine and the BCG vaccine. Implications for the Expanded Programme on Immunization. [phd-thesis]. University of Copenhagen; 1999.
Ref ID: 872

(44) Garly M-L, Bale C, Martins CL, Monteiro M, George E, Kidd M et al. Measles antibody responses after early two dose trials in Guinea-Bissau with Edmonston-Zagreb and Schwarz standard-titre measles vaccine: better antibody increase from booster dose of the Edmonston-Zagreb vaccine. Vaccine 2001; 19(15-16):1951-1959.
Ref ID: 1132

(45) Garly M-L, Aaby P. The challenge of improving the efficacy of measles vaccine. Acta Trop 2003; 85(1):1-17.
Ref ID: 1595
